# A Novel Kalman Filter Design and Analysis Method Considering Observability and Dominance Properties of Measurands Applied to Vehicle State Estimation

**DOI:** 10.3390/s21144750

**Published:** 2021-07-12

**Authors:** Julian Ruggaber, Jonathan Brembeck

**Affiliations:** Institute of System Dynamics and Control, Robotics and Mechatronics Center, German Aerospace Center (DLR), 82234 Weßling, Germany; jonathan.brembeck@dlr.de

**Keywords:** Kalman filter, estimator design, nonlinear state estimation, nonlinear observability, tire-road friction coefficient, vehicle dynamics, vehicle state estimation

## Abstract

In Kalman filter design, the filter algorithm and prediction model design are the most discussed topics in research. Another fundamental but less investigated issue is the careful selection of measurands and their contribution to the estimation problem. This is often done purely on the basis of empirical values or by experiments. This paper presents a novel holistic method to design and assess Kalman filters in an automated way and to perform their analysis based on quantifiable parameters. The optimal filter parameters are computed with the help of a nonlinear optimization algorithm. To determine and analyze an optimal filter design, two novel quantitative nonlinear observability measures are presented along with a method to quantify the dominance contribution of a measurand to an estimate. As a result, different filter configurations can be specifically investigated and compared with respect to the selection of measurands and their influence on the estimation. An unscented Kalman filter algorithm is used to demonstrate the method’s capabilities to design and analyze the estimation problem parameters. For this purpose, an example of a vehicle state estimation with a focus on the tire-road friction coefficient is used, which represents a challenging problem for classical analysis and filter parameterization.

## 1. Introduction

The degree of automation and technical support for humans has increased rapidly in recent years. The basic requirement for any control system is the existence of measurable control variables. If they cannot be measured directly due to technical or economic reasons, state estimators are needed. Thus, state estimation forms the backbone of most modern control problems and is required for their implementation. One proven method for state estimation is Kalman filtering. The Kalman filter is an algorithm that provides optimal estimates for the states of a dynamical system sequentially in time. The disturbances in the underlying mathematical model of the system and measurement equations are assumed to be white noise [1]. This established and widely used method has been known and applied for more than 60 years now. Many different modifications of this method have been developed. Nevertheless, there is still no simple procedure for an optimal design or parametrization of a Kalman filter. This task is often executed by experienced control engineers based on empirical knowledge or by trial and error experiments through Monte-Carlo simulations, see, e.g., [2].

One of the most fundamental requirements for an estimation problem is its observability. This means that the estimated states, which are reconstructed, have to be contained in the measurements and uniquely extracted from them. In order to parametrize a filter, the covariance matrices need to be determined (when dealing with other filter types such as, e.g., an unscented Kalman filter, there might be even more parameters). This leads to the crucial question for the filter design as to which measurands should actually be used and how they influence the estimation problem. In many cases, the properties of the measurands for the estimation problem are only evaluated by simple plausibility checks. For instance, when estimating the state-of-charge (see e.g., [3]) of a battery, it is evident that the battery voltage should be used as a measurand. But how would other measurands affect the observability, and might they be even more dominant? Issues like these are addressed in this paper in a quantifiable way with the help of the novel design and analysis method presented below.

### 1.1. State of the Art

The authors of [4] present an automated method that allows the determination of proper Kalman filter parameterization. To evaluate the estimation accuracy, a performance index, which is analytically related to the filter parameterization, is introduced. By minimizing this index, optimal parameterization can be calculated. In [5], a relationship between the performance values and filter parameterization is investigated. However, neither an exact relationship between these values nor a calculation rule is presented. A similar relationship is presented in [6] using the example of a video tracking system. In [7], a method is shown where the filter is considered as a control system, thus allowing corresponding tuning criteria to be derived. In [8], the filter parameters are computed via optimization based on a genetic algorithm. The authors in [9] use Bayesian optimization for this purpose. Instead of minimizing a cost function, this approach tries to maximize the probability of improving the current best solution. In [10], a two-step method is presented in which particle swarm optimization (PSO) is used to tune both the filter parameterization and prediction model parameters.

**Figure 1:**
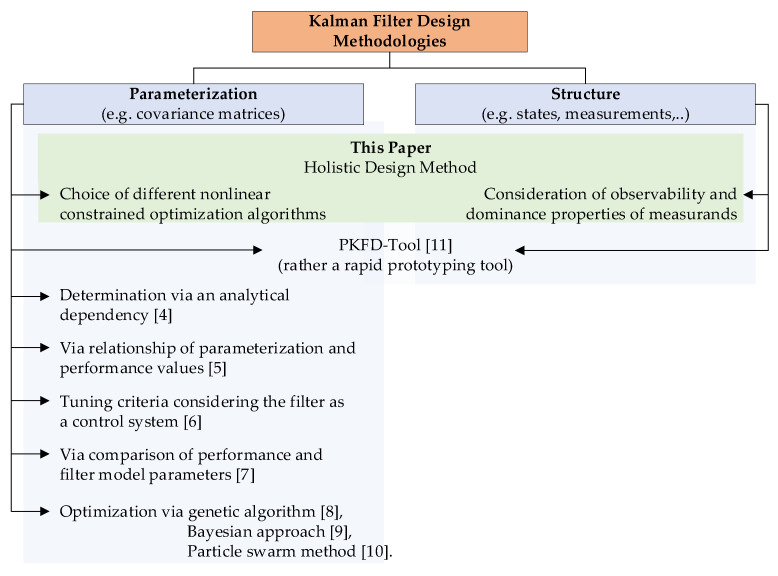
Overview of Kalman filter design methodologies.

Besides filter parameterization, the filter structure, i.e., the selection of state variables and measurands, is perhaps an even more important design issue. However, the methods mentioned so far cannot help to solve this problem. The so-called Programmable Kalman Filter Design Tool (PKFD) is shown in [11]. This tool provides both an optimal parameterization (system noise, measurement noise, and initial estimation error covariance matrices) and an optimal filter structure. Nevertheless, the tool rather serves as a rapid prototyping environment, allowing different Kalman filter setups to be compared, but not giving a precise analysis of the properties. Figure 1 shows a classification of the Kalman filter design methodologies mentioned above.

### 1.2. Contribution of This Paper

In the approaches mentioned so far, the design focus lies solely on the filter parameterization, but not on its structure. To fill this research gap, both design issues are addressed and evaluated in detail by the holistic method elaborated in this paper.

Using a novel state-specific nonlinear quantitative observability measure, the current “observability accuracy” can be determined at any time in an estimation problem. Additionally, a new “dominance analysis” method allows the percentage contribution of each measurand to an individual state to be calculated. Based on this knowledge about the observability and dominance properties of all possible measurands, different filter setups can be compared with each other. As a result, the benefit of a measurand can be evaluated in comparison with the effort required to provide it in reality. Furthermore, the filter covariance matrices (as well as other possible filter parameters) are determined by a nonlinear optimization algorithm considering the filter self-diagnosis and resulting in a minimum estimation error.

In summary, the method enables a holistic filter design and provides quantitative criteria for an optimal filter configuration, namely:

- Optimal filter parameterization using a nonlinear constrained optimization algorithm.
- Optimal filter structure using a quantitative nonlinear state-specific observability measure and a dominance analysis to evaluate the influence of the measurands’ properties on the estimation problem.

This novel method is universally applicable. As an example, this paper considers its application to a vehicle state estimation problem using an unscented Kalman filter (UKF).

## 2. Fundamentals of State Estimation

A well-proven method for state estimation is Kalman filtering. Here, the system modeling, as well as the measurements, are described by their statistical characteristics, and an optimal estimation [1] is performed by an iterative procedure (prediction and measurement update). This section closely follows [3,12]; the interested reader is referred to these sources for more detail.

### 2.1. System Description

For many control system tasks, the plant model to be used in state estimation is naturally described as a nonlinear continuous-time state-space system:

(1) $$\begin{array}{l} \dot{x}=f\left(x,u\right),\\ y=h\left(x\right),\\ u\left(t\right)\in {\mathbb{R}}^{s\times 1},\;x\left(t\right)\in {\mathbb{R}}^{n\times 1},\;y\left(t\right)\in {\mathbb{R}}^{m\times 1},\;t\in \mathbb{R}. \end{array}$$

where t is the time, u(t) is the vector of inputs, x(t) is the vector of states, and y(t) is the vector of outputs. In a sampled data system (e.g., a microcontroller) the continuous-time model representation in Equation (1) cannot be used directly. Instead, a time-discrete representation is needed, and therefore, the time-discrete transformation of Equation (1) with additive Gaussian noise is used in the sequel:

(2) $$\begin{array}{ll} x_{k} & =f_{k|k-1}\left(x_{k-1},u_{k-1}\right)+w_{k-1},\\ y_{k} & =h\left(x_{k}\right)+v_{k},\\ & w_{k}\;\sim \;N\left(0,Q_{k}\right),\\ & v_{k}\;\sim \;N\left(0,R_{k}\right). \end{array}$$

Here, $t_{k}$ is the k-th sample time instant of a periodically sampled data system with $u_{k}=u\left(t_{k}\right)$, $x_{k}=x\left(t_{k}\right)$ and $y_{k}=y\left(t_{k}\right)$. The vectors $w_{k}$ and $v_{k}$ represent zero biased Gaussian white noise. The covariance matrices $Q_{k}$ and $R_{k}$ are defined as $E\left(w_{k}w_{j}^{T}\right)=Q_{k}\delta_{k-j}$ and $E\left(v_{k}v_{j}^{T}\right)=R_{k}\delta_{k-j}$, where $\delta_{k-j}$ is the Kronecker delta function; that is, $\delta_{k-j}=1$ if k=j, and $\delta_{k-j}=0$ if k≠j. Note that as a simplification, $Q_{k}$ is assumed to be a diagonal matrix, but in general, it may contain cross-correlation terms between the states (see, e.g., [13]). The operator E(·) calculates the expected value of a random variable [13]. The notation $w_{k}\sim N\left(0,Q_{k}\right)$ indicates that $w_{k}$ is a Gaussian random variable with a mean vector of 0 and a covariance matrix of $Q_{k}=\mathrm{diag}\left(\sigma_{1..n_{x}}^{2}\right)$, with the standard deviation σ.

### 2.2. Constrained State Estimation

Similar to an optimization problem, a state estimation problem can be advantageously simplified by introducing constraints on the possible solution space, e.g., by physical limits, thus reducing the complexity of the problem. There are several methods dealing with constraints for state estimators, see [14]. As they are beyond the scope of this paper, they will not be discussed in any further detail here. A summary of the methods with their advantages and disadvantages can be found in [12]. In the present paper, two linear constraints are applied (a lower and an upper bound for the maximum tire-road friction coefficient (TRFC)), see Section 4.1.3. For this purpose, the method described in [12] (p. 79) is used. By means of a root-finding problem, the feasible state variables are determined. For linear constraints, as is the case for this paper, the method provides an optimal solution to the problem.

### 2.3. DLR Kalman Filter Estimation Framework

For this research work, we use and extend the DLR Kalman Filter estimation framework [12], which uses prediction models based on continuous-time Modelica models and automatically generates model-based nonlinear state estimators. The approach is based on an extended FMI (Functional Mockup Interface) 2.0 co-simulation interface [15] that interacts with the state estimation algorithms implemented in the DLR Kalman Filter Library [16]. Starting from a multi-physical Modelica model (continuous-time, usually nonlinear), a nonlinear prediction model is automatically generated in the form of a sampled data system (cf. Equation (2)). The framework employs an intelligent separation of the model (encapsulated in a standardized FMI 2.0 for co-simulation [15]) and the estimation algorithm by utilizing modern computer technologies and recent developments in the Modelica language [17]. They enable automated discretization, integration, and derivative calculation of an object-oriented equation-based prediction model. The FMI defines a standardized interface to be used in computer simulations to develop complex cyber-physical systems. The following estimation algorithms are implemented reliably and efficiently in the DLR Kalman Filter Library: EKF, EKF SR (EKF square-root), EKF UD (EKF UD-decomposition), UKF (unscented Kalman filter), and UKF SR (UKF square-root), see [1,18]. Additionally, there are modified algorithms for parameter estimation as well as an extension to nonlinear moving horizon estimation (MHE) using a fast nonlinear gradient descent search, as is presented in [12]. Recently, the library’s features were extended to meet the requirements of embedded targets. This was part of the ITEA EMPHYSIS project in which a new embedded FMI (eFMI) standard specification was designed [19].

## 3. Design Method for Kalman Filters

The structural filter design is based on the observability and dominance analysis of measurands. This section starts with the introduction of three different observability measures for nonlinear systems. In addition to the probably best-known one-rank analysis of the observability matrix, two new approaches for a quantitative statement about observability are presented. Next, these three methods are compared by means of an illustrative example. Moreover, the advantages of the two newly developed observability measures are discussed. Afterward, a new approach—the so-called dominance analysis—is described to quantify the contribution of a measurand to the estimation. Finally, the holistic filter design method is shown, which consists of the observability and dominance analysis embedded in an optimization framework.

### 3.1. Nonlinear Observability Measures

The observability problem together with its counterpart, the controllability problem, are important parts of the systems theory. Especially when designing observers, observability plays a key role. In this section, three methods for nonlinear observability analysis are presented. For reasons of clarity, the time dependence is not explicitly indicated by an argument for the respective variables.

The observability of a dynamic system is a property that is independent of the estimation method, but is solely determined by the

- **structure** of the problem, i.e., which measurements are available and how they are linked to the states (measurement equation).
- **excitation** of the system by the input u, which has to take a minimum value (*persistence of excitation*).

#### 3.1.1. Observability via Rank Condition

Probably the best-known method for the observability analysis of nonlinear systems is the rank investigation of the observability matrix. This matrix is built using the n−1 Lie derivatives $y,\;\dot{y},\;\ldots y^{\left(n-1\right)}$ [13], whereby y indicates the system output from Equation (1). Hence, the nonlinear observability matrix is:

(3) $$\underset{≔Y}{\underset{︸}{\left[\begin{array}{c} y\\ \dot{y}\\ \ddot{y}\\ \vdots \\ y^{\left(n-1\right)} \end{array}\right]}}=q\left(x,u,\dot{u},\ldots ,u^{\left(n-1\right)}\right).$$

The system is globally observable if a unique inverse function of Equation (3) can be found, which occurs, however, possible only in very rare cases in practical applications. Instead, Equation (3) can be linearized along the reference trajectories $\zeta_{\mathrm{ref}}=\left[\begin{array}{ccccc} x & u & \dot{u} & \cdots & u^{\left(n-1\right)} \end{array}\right]$

(4) $$O_{M}={\frac{\partial q\left(x,u,\dot{u},\ldots ,u^{\left(n-1\right)}\right)}{\partial x}|}_{\zeta_{\mathrm{ref}}},$$

making the observability investigation at certain points possible. A linear system of equations is obtained:

(5) $$\Delta Y=O_{M}\cdot \Delta x.$$

If $O_{M}$ is invertible, i.e., has full rank *n*, the states x can be obtained via $\Delta x=O_{M}^{-1}\cdot \Delta Y$. The nonlinear system in Equation (1) is called weakly observable for a given domain if the observability matrix for this domain does not have a rank loss, i.e., if it holds

(6) $$\mathrm{rank}\left(O_{M}\right)=n.$$

The criterion of a rank loss provides only a binary statement about the observability and is numerically highly sensitive. Quantitative information, regarding how good or bad the observability is, is not given. After the presented classical rank-based observability analysis, the next two sections introduce two new observability measures allowing a quantitative statement about the observability.

#### 3.1.2. Quantitative Observability Measure Considering Numerical Condition Number

The crucial question for the observability quantification is how far $O_{M}$ is away from a rank loss. The geometric interpretation of the numerical condition number κ(·) of a matrix provides an answer to this question. Namely, the reciprocal condition number indicates the relative distance (w.r.t. the Euclidean norm) of a non-singular matrix to its nearest singular matrix [20] (p. 242):

(7) $$min\left\{\frac{{\left\|\Delta O_{M,s}\right\|}_{2}}{{\left\|O_{M}\right\|}_{2}}\;:O_{M}+\Delta O_{M,s}\;\mathrm{is}\;\sin\mathrm{gular}\right\}=\frac{1}{\kappa \left(O_{M}\right)}.$$

The “distance to the singularity”, which can be quantified via $\kappa \left(O_{M}\right)$, corresponds to the “distance to a rank loss” of $O_{M}$ and can, therefore, be interpreted as a quantitative observability measure.

**Figure 2:**
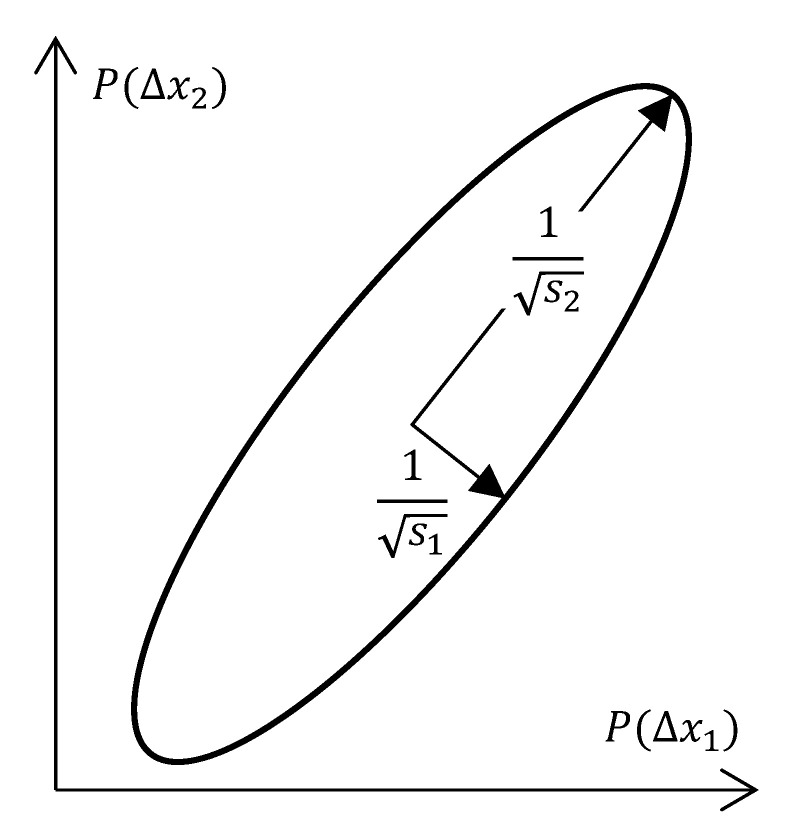
Confidence covariance ellipse.

The relationship between the numerical condition number and the observability properties can be clearly shown by considering the covariance-error-ellipsoid (a.k.a. confidence ellipsoid) of Equation (5). In Figure 2, this is shown as an example of a system with order n=2.

The length of the orthogonal half-axes of the covariance ellipsoid corresponds to the inverse root of the singular values ${\sqrt{s_{i}}}^{-1}$ of $O_{M}$ [21] (p. 692ff.). The numerical condition number is the ratio of the largest singular value to the smallest one [22]

(8) $$\kappa \left(O_{M}\right)=\frac{s_{\max}}{s_{\min}},$$

providing a statement about the shape of the ellipsoid. For n=2, large values of the condition number mean a narrow ellipse. This means that existing small uncertainties of one state lead to large uncertainties of another state, which implies bad observability. A good condition number of $O_{M}$, i.e., small values of $\kappa \left(O_{M}\right)$, is equivalent to good observability.

One main disadvantage of the two methods presented above is the high computational effort required for the calculation of the Lie derivatives in the observability matrix. This effort grows with the numbers of states and outputs. Furthermore, the statements about observability are valid only for the whole system, but not for single states. This means the two presented measures show a loss of observability, even though only a single state is unobservable. Often, not all states are of equal interest, wherefore a state-specific quantitative observability measure is presented hereinafter.

#### 3.1.3. State-Specific Quantitative Observability Measure Using Weighted Least Squares

The basic idea of the state-specific observability measure introduced in this section is assessment of the observability via the weighted least squares (WLS) solution for the states applied to the linearized measurement equation.

If the output in Equation (1) is linearized along the reference states $x_{\mathrm{ref}}$ (denoted as the ground truth states; since the observability is independent of the used estimation algorithm and depends only on the underlying system structure and excitations (see Section 3.1), the linearization has to be performed around the reference states, but not around the estimated ones), the measurement sensitivity matrix $H_{\mathrm{ref}}$ is obtained as

(9) $$H_{\mathrm{ref}}={\frac{\partial }{\partial x}h\left(x,u\right)|}_{x_{\mathrm{ref}}},$$

and the linear measurement equation implies

(10) $$z_{\mathrm{lin}}=H_{\mathrm{ref}}\cdot \Delta x_{\mathrm{ref}}+v.$$

**Remark:** If an extended Kalman filter is used for the estimation, the measurement sensitivity matrix advantageously results from the filter algorithm as a by-product. However, instead of linearizing around the estimated states, the linearization has to be performed around the reference states.

Under the condition that at least as many measurements are available as states, i.e., in case of m≥n, and rank($H_{\mathrm{ref}}$) =n, Equation (10) is overdetermined and thus solvable. This means that with known measurements z, Equation (10) can be solved directly to find the states using a curve-fitting approach. A suitable method for this is a least-square approach. The measurements need to be weighted by the inverse measurement noise covariance matrix R, so that a weighted least square (WLS) problem formulation [23] can be used, leading to

(11) $$x_{\mathrm{WLS}}=\underset{≔P_{\mathrm{obs}}}{\underset{︸}{{\left(H_{\mathrm{ref}}^{T}R^{-1}H_{\mathrm{ref}}\right)}^{-1}}}H_{\mathrm{ref}}^{T}R^{-1}z$$

with the matrix $P_{\mathrm{obs}}\in {\mathbb{R}}^{n\times n}$.

**Remark:** The calculation of $P_{\mathrm{obs}}$ via a singular value decomposition is computationally demanding (see [22]) and can be calculated by a QR decomposition in a more efficient way [12] (p. 39ff).

$P_{\mathrm{obs}}$ is the covariance matrix of the WLS estimator and indicates the impact of the uncertainty of the measurements z on the states $x_{\mathrm{WLS}}$. Exactly this quantification, namely, how well the states can be reconstructed from the measurements, corresponds to the definition of observability. Thus, the diagonal entries of $P_{\mathrm{obs}}$

(12) $$\mathrm{diag}(P_{\mathrm{obs}})=\left[\begin{array}{ccc} \sigma_{\mathrm{obs},x_{1}}^{2} & \cdots & \sigma_{\mathrm{obs},x_{n}}^{2} \end{array}\right]$$

represent a quantitative observability measure. The covariance entries $\sigma_{\mathrm{obs},x_{i}}^{2}$ indicate the current observability of the $i^{th}$ state. Large variance values imply bad observability, while small values imply it is good. Compared to classical rank loss-based approaches, the presented method allows a state-specific quantitative statement about the observability. Furthermore, the covariance entries $\sigma_{\mathrm{obs},x_{i}}^{2}$ have the same physical units as the states, making their physical interpretability possible. Due to the simple WLS formulation, the method can be executed quickly and computationally efficiently.

#### 3.1.4. Comparison of Observability Measures

This section compares the three observability measures using an illustrative example of the tire road friction coefficient (TRFC) estimation. For this purpose, the nonlinear two-track model from Section 4.1.2 is exploited (its exact knowledge is not yet required to understand the example). Since this section is only aimed at comparing the observability measures, the reference model corresponds to the filter prediction model. Sinusoidal steering with a constant vehicle speed is simulated as a maneuver. This implies that the system is excited solely by the lateral acceleration $a_{y}^{C}$. Therefore, observable and non-observable phases can be specifically generated for the TRFC $\mu_{\max}$.

**Figure 3:**
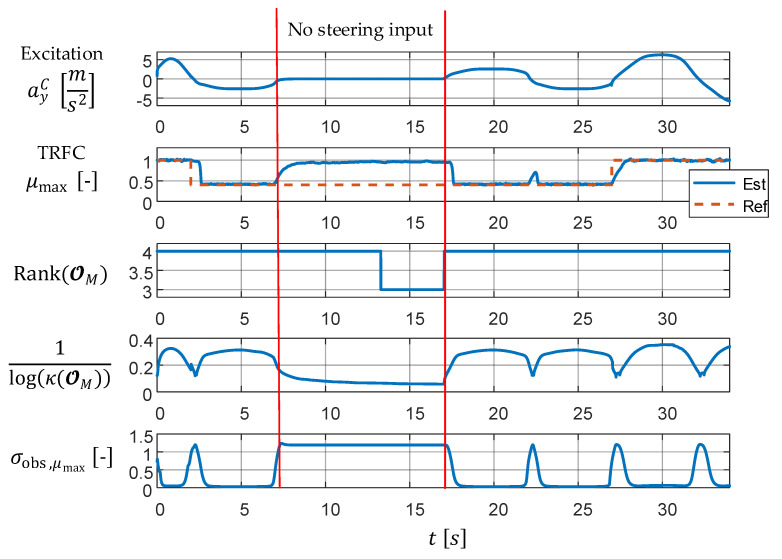
Comparison of the three observability measures for the simple $\mu_{\max}$ (TRFC) estimation example.

Figure 3 shows a maneuver range without excitation between t=7 s and t=17 s, in which no acceleration, braking, or steering are present. It is obvious that in the area where the vehicle simply rolls straight ahead, the TRFC is not observable. 

At the very top of Figure 3, the excitation by the lateral acceleration $a_{y}^{C}$ is given. The plots below show the TRFC $\mu_{\max}$, its estimation, and the following three observability measures:

- The rank of $O_{M}$, which returns only a binary yes/no-observability assessment.
- The reciprocal numerical condition number of $O_{M}$ (due to large condition numbers, the logarithm base 10 is taken for reasons of clarity). Small values correspond to poor observability, whereas large values indicate good observability.
- The standard deviation of the TRFC $\sigma_{\mathrm{obs},\mu_{\max}}$ using the WLS approach. Large values imply high uncertainty, i.e., poor observability, and small values vice versa. The specified uncertainty has the same physical unit as the TRFC, making its direct and clear interpretation possible.

The jump of $\mu_{\max}$ to a low friction value can be correctly estimated shortly after its occurrence at t=2 s if there is sufficient excitation. From t=7 s on, there is no excitation anymore, so the observability is lost. Therefore, the estimated TRFC follows its first order lag (PT1) behavior given in the system model and tends towards the set value of $\mu_{\max}=1$.

The rank of the observability matrix decreases until t=13 s, indicating a loss of observability with a delay of 6 s. The two quantitative measures indicate observability loss immediately after its occurrence. According to the WLS approach, the current uncertainty is $\sigma_{\mathrm{obs},\mu_{\max}}\approx 1.2$, which corresponds to the total loss of observability. From t=17 s on, there is again sufficient excitation, so that all three measures again show observability, and the TRFC can be estimated correctly. In the two quantitative measures, it can also be clearly seen that at each zero crossing of $a_{y}^{C},$ the observability becomes poor, as in this case, there is no excitation for a short time.

This example shows the advantages of the two introduced novel observability measures. While the well-known rank loss-based criterion indicates the loss of observability with insufficient accuracy, the measures $\kappa \left(O_{M}\right)$ and $\sigma_{\mathrm{obs},x}$ immediately provide a quantified statement about how good or bad the current observability is. The condition number $\kappa \left(O_{M}\right)$ only provides a statement about the whole system. However, in many cases, some states are observable, while others are not. Since the focus in this paper is directed towards the TRFC, i.e., the state $\mu_{\max}$, the state-specific observability criterion $\sigma_{\mathrm{obs},x_{i}}$ is used below.

### 3.2. Dominance Measure: Individual Contribution of Measurands to Estimated States

In the structural design of a Kalman filter, an essential question is which measurands contribute to the estimation of a state and how much. A simple and practical method to determine this contribution can be realized by considering the equation for the filter measurement update:

(13) $${\hat{x}}_{k}^{+}={\hat{x}}_{k}^{-}+\underset{≔m_{k}}{\underset{︸}{K_{k}\cdot \underset{≔\;d_{k}}{\underset{︸}{\left(z_{k}-{\hat{h}}_{k}^{-}\right)}}}},$$

where $d_{k}\in {\mathbb{R}}^{m\times 1}$ is called the *innovation*. The second summand $m_{k}\in {\mathbb{R}}^{n\times 1}$ of Equation (13) indicates an incremental contribution of each measurand to the corresponding state:

(14) $$m_{k}=\underset{≔K_{k}}{\underset{︸}{\left[\begin{array}{c} k_{x_{1}}\\ k_{x_{2}}\\ \vdots \\ k_{x_{n}} \end{array}\right]}}\cdot \left[\begin{array}{c} d_{k,1}\\ d_{k,2}\\ \vdots \\ d_{k,m} \end{array}\right].$$

The row vectors $k_{x_{l}}\in {\mathbb{R}}^{1\times m},l\in \left\{1,\ldots ,n\right\}$ of the Kalman gain matrix $K_{k}\in {\mathbb{R}}^{n\times m}$ contain the m gains of the innovation $d_{k}$ for the $l^{th}$ state. Through the element-wise multiplication

(15) $$\gamma_{k,x_{l}}=k_{x_{l}}⊙d_{k}^{T},$$

the vector $\gamma_{k,x_{l}}\in {\mathbb{R}}^{1\times m}$ is obtained, whose elements are the proportion of the measurements in the estimation of the $l^{th}$ state. For the investigation of a discrete time interval $k\in \left[k_{0},k_{\mathrm{end}}\right]$, the absolute values of the measurements can be summed up. For better comparability, normalization is performed to quantify the relative shares in the overall estimation:

(16) $${\bar{\gamma }}_{x_{l}}=\underset{≔p}{\underset{︸}{{\left[\begin{array}{ccc} \overset{k_{\mathrm{end}}}{\underset{k=k_{0}}{\sum }}\left|\gamma_{k,x_{l},1}\right| & \cdots & \overset{k_{\mathrm{end}}}{\underset{k=k_{0}}{\sum }}\left|\gamma_{k,x_{l},m}\right| \end{array}\right]}^{T}\cdot 𝟙\left(\sigma_{k,\mathrm{obs},x_{l}}<\bar{\sigma_{\mathrm{obs},x_{l}}}\right)}}\cdot \frac{1}{\sum_{j=1}^{m}p_{j}}.$$

Analysis of the measurands’ influence makes sense only for time points in which the state is observable. Due to the indicator function 𝟙(·) defined as

(17) $$𝟙\left(x\right)=\{\begin{array}{c} 0,\;\mathrm{if}\;x\leq 0,\\ 1,\;\mathrm{if}\;x>0, \end{array}$$

only those time points k are considered, in which the observability measure for the l-th state $\sigma_{k,\mathrm{obs},x_{l}}$ does not exceed a fixed upper bound $\bar{\sigma_{\mathrm{obs},x_{l}}}$. This results in a scalar value for each measurand. The larger the value, the more dominant the sensor is in the overall estimation. Hereinafter, the method is, therefore, referred to as dominance analysis.

### 3.3. Novel Holistic Method for Kalman Filter Design

Based on the concepts described in the previous sections, a holistic method is presented hereinafter to optimally design a Kalman filter with respect to structure and parameterization.

**Optimal filter structure:** To tackle the fundamental issue of appropriate measurands’ selection, their respective influence on the estimation problem has to be quantified. On the one hand, the state-individual quantitative observability measure presented in Section 3.1.3 is used to analyze the properties of a measurand with respect to observability. On the other hand, the dominance analysis according to Section 3.2 allows the individual contribution of each measurand to the estimation to be evaluated. This enables the identification of sensor variables with a high or low information content and to accordingly adjust their selection.

**Optimal filter parameterization**: The DLR’s Multi-Objective Parameter Synthesis (MOPS) optimization tool [24] is used to determine the optimal filter parameters. *MOPS* provides a variety of optimization methods. For the present design method, a pattern search approach is exploited, being able to deal with constrained nonlinear problems.

**Figure 4:**
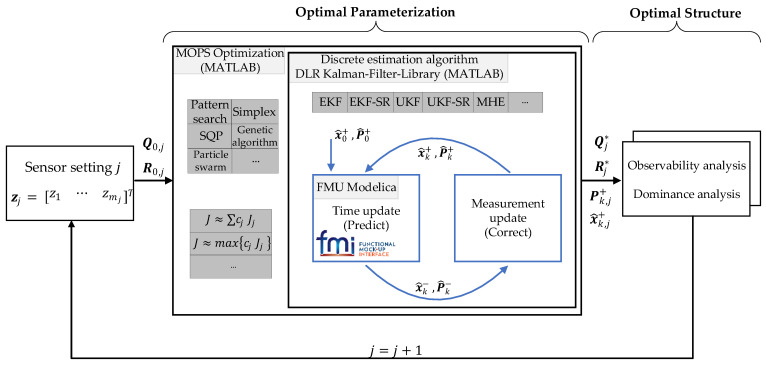
Overview of the Kalman filter design and analysis method.

The holistic Kalman filter design method is an iterative process combining the concepts presented above as shown in Figure 4.

Starting from an arbitrary sensor configuration j, j∈ℕ, the system and measurement noise covariance matrices $Q_{j}$ and $R_{j}$, as well as the UKF parameters ${\left(\alpha ,\;\beta ,\;\kappa \right)}_{j,\mathrm{UKF}},$ are determined with the help of *MOPS*. Considering the requirement for an optimal tracking behavior with the estimation error $\epsilon_{x}$ to be minimized, the optimization problem, i.e., the cost function, can be formulated as

(18) $$J=\underset{\begin{array}{c} Q_{j},\;R_{j},{\left(\alpha ,\;\beta ,\;\kappa \right)}_{j,\mathrm{UKF}} \end{array}}{\min}\left\{c_{l}\sum_{k}{\left(\underset{≔\epsilon_{x,k}}{\underset{︸}{x_{k}-{\hat{x}}_{k}^{+}}}\right)}^{2}\right\},$$

s.t. inequality constraints

(19) $$\left|\epsilon_{x,k,l}\right|-\sqrt{\mathrm{diag}\left(P_{k}^{+}\right)}\leq 0,$$

where $c_{l},\;l\;\epsilon \;\left\{1,\ldots ,n\right\}$, is a specific weight factor for each state estimation error. The inequality constraint considers the filter self-diagnostics, according to which the filter only works correctly if the estimation errors lie within the confidence interval, i.e., if the standard deviation of the estimation errors is $\pm \sqrt{\mathrm{diag}\left(P_{k}^{+}\right)}$. According to the Gaussian distribution, ≈68% of all estimation errors lie within the confidence interval corresponding to $\pm \sqrt{\mathrm{diag}\left(P_{k}^{+}\right)}$. Thus, about 30% of the values are not considered, although the filter would work correctly. For that reason, the constraints appear to be rather restrictive.

The filter optimally parameterized with *MOPS* can then be evaluated for a maneuver by means of the observability analysis with the criterion $\sigma_{\mathrm{obs},\mu_{\max}}$ presented in Section 3.1.3, as well as the dominance analysis presented in Section 3.2. This loop can be run through for a filter configuration j. By comparing different sensor settings, a quantitative statement of how much a sensor contributes to the observability (observability analysis) and to the estimation (dominance analysis) can be made. Thus, the effort to provide a measurand, in reality, can be compared to its benefit for the estimation.

## 4. Vehicle State Estimator

The presented universally applicable design and analysis method is exemplarily applied to a vehicle state estimation problem using an unscented Kalman filter (UKF). The vehicle considered in this paper is the ROboMObil (ROMO)—an innovative robotic electric vehicle concept developed at the DLR Robotics and Mechatronics Center. The design of the ROMO is based on the so-called “wheel robot” concept [25] with all wheel by-wire steering capabilities, where the drivetrain, brakes, steering system, spring, and dampers are integrated into each of the four wheels. The dissemination of electric vehicles in the automotive market results in a variety of estimation problems related, e.g., to the battery state [3] as well as to the vehicle dynamics, see, e.g. [26].

The most important variables for describing driving stability are probably the vehicle side-slip angle as a measure of the current vehicle stability, and the maximum coefficient of friction between the tire and the road (TRFC) as a measure of the stability limit. Although the vehicle side-slip angle can be directly measured by a high-precision IMU (inertial measurement unit) or an optical road sensor [12], it is only estimated in production vehicles due to the high costs of such sensors. Methods for estimating the TRFC have been intensely researched in science and technology for decades [27,28]. The reconstruction of the TRFC from the measurements is quite complex since a certain excitation threshold has to be exceeded in order to distinguish between the different coefficients of friction. Knowledge about the TRFC is of paramount importance for manned driving since the road conditions are often underestimated even by experienced drivers. At the same time, the TRFC also plays a key role for autonomous driving functions because computers, unlike human drivers, have no intuition that, for example, the speed should be reduced on a wet road covered with leaves. In addition to the vehicle side-slip angle and the TRFC, the vehicle velocity over ground and the vehicle yaw rate are also estimated. However, the focus will lie on the TRFC due to its growing importance and the complexity of its estimation.

### 4.1. Vehicle Models

In this section, two vehicle models are presented. The first one is a detailed and highly accurate full vehicle model, which serves as the reference. Since the model is simulated only once per maneuver to generate the reference data, the high effort required for this computation is acceptable. The second model is the filter prediction model, which is permanently simulated during the design and analysis process. Therefore, this model has to be computationally more efficient than the first one.

#### 4.1.1. High Fidelity Reference Model

The full vehicle model is a detailed, high-precision validated multiphysics Modelica model, which, as a reference, represents real vehicle behavior in a very accurate manner [29]. In addition to the vehicle model, there is an extensive environmental model in which parameters such as the road gradient, the ambient temperature, or the coefficient of friction between tire and road can be defined. Moreover, there is a driver model that allows individual trajectories to be driven in addition to predefined standard maneuvers. The chassis is a kinematic multi-body model. Pacejka’s Magic Formula 5.2 [30] is used as the tire model. The spring and damper characteristics are described by nonlinear characteristic curves. The overall model has over 100 states and a nonlinear system of equations with a dimension >500.

#### 4.1.2. Filter Prediction Model

**Figure 5:**
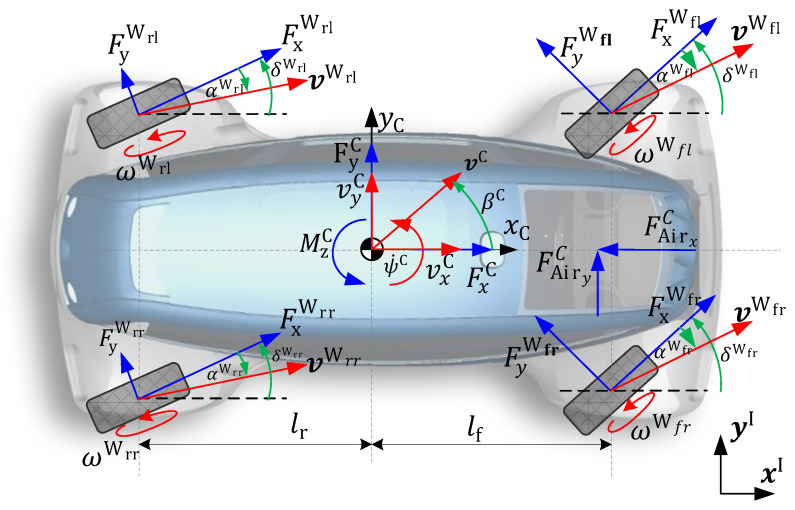
Vehicle dynamics quantities of the nonlinear two-track model based on [26].

A nonlinear two-track model is used for the prediction model in the filter, see Figure 5. The quantities marked by ${\left(\cdot \right)}^{C}$ are expressed in the car coordinate system with an origin in the center of gravity (CoG), while those indicated by ${\left(\cdot \right)}^{W_{i}}$ are in the $i^{th}$ wheel robot coordinate system. The vehicle state is described by four states: side-slip angle $\beta^{C}$, vehicle velocity over ground $v^{C}$, maximum tire-road friction coefficient $\mu_{\max}$, and yaw rate ${\dot{\psi }}^{C}$. The equations of motion are thus obtained as:

(20) $${\dot{\beta }}^{C}=\frac{1}{v^{C}}\left(-a_{x}^{C}\sin\left(\beta^{C}\right)+a_{y}^{C}\cos\left(\beta^{C}\right)\right)-{\dot{\psi }}^{C},$$

(21) $${\dot{v}}^{C}=a_{x}^{C}\cos\left(\beta^{C}\right)+a_{y}^{C}\sin\left(\beta^{C}\right),$$

(22) $${\dot{\mu }}_{\max}=\frac{1}{T_{\mu }}\left(1-\mu_{\max}\right),$$

(23) $${\ddot{\psi }}^{C}=\frac{1}{I_{z}}M_{z}^{C}.$$

For the tire model representing the main nonlinearity in the vehicle model, a slightly simplified version of Pacejka’s Magic Formula 5.2 [30] is used. The detailed tire model can be found in Appendix B. The maximum tire-road friction coefficient TRFC, which is tire-specific in reality, is described as a whole-vehicle equal parameter in this paper. The TRFC is a parameter in the tire model, which is often described in the literature as ${\dot{\mu }}_{\max}=0$ (representing a random walk process under the presence of white noise). Another possibility is to model ${\dot{\mu }}_{\max}$ as a first-order filter (PT1). When the excitation is insufficient, the friction value would converge to a predetermined value [31], e.g., $\mu_{\max}=1$ (see Equation (22) with $T_{\mu }=1s$), which corresponds to a high friction value. This state description is also called artificial stabilization [32]. An additional benefit of the PT1 formulation is its anti-windup effect in a constrained estimation. As presented in the next section, the TRFC is constrained in the measurement update to a maximum value of 1. In a random walk state description, for example, this would cause the integrators to accumulate the TRFC even during saturation (wind-up). The system would need some iteration steps to “unload” again. A detailed presentation of the model equations complementing Equations (20)–(23) can be found in Appendix A.

#### 4.1.3. Constraint of the Tire Road Friction Coefficient

**Figure 6:**
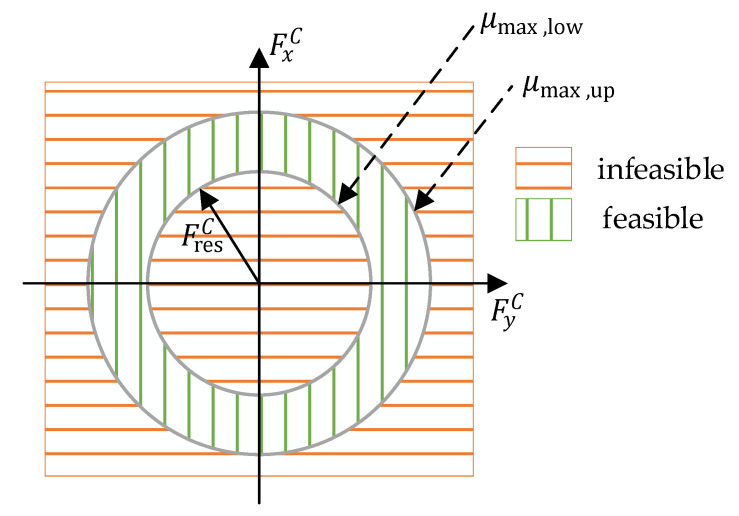
TRFC constraint based on the Kamm circle and its limits.

For the estimation problem presented here, it is useful to integrate prior physical knowledge about the state TRFC $\mu_{\max}$ into the estimation via the constraints. The currently utilized frictional force between the tire and the road, i.e., the instantaneous friction value $\mu_{\mathrm{act}}$, is calculated via the relationship of the Kamm circle (see Figure 6):

(24) $$\mu_{\mathrm{act}}=\frac{F_{\mathrm{res}}^{C}}{\sum_{i=1}^{4}F_{z}^{W_{i}}}=\frac{1}{g}\sqrt{{a_{x}^{C}}^{2}+{a_{y}^{C}}^{2}}.$$

Obviously, the measured longitudinal and lateral vehicle accelerations can be exploited to approximate the currently utilized friction value. With the assumption that the vehicle is in a stable state of motion, the friction value at time step k for the dynamic lower bound is determined to be:

(25) $$\mu_{\max,\mathrm{low},k}\geq \mu_{\mathrm{act},k}.$$

For the maximum possible TRFC, a value of 1 is assumed (depending on the tire type, however, values for $\mu_{\max}$ up to 1.2 are also possible on dry surfaces [28]). For the upper bound, this provides:

(26) $$\mu_{\max,\mathrm{up},k}\leq 1.$$.

The presented constraints primarily concern the Kalman filter measurement update step. The values of the prediction step are also indirectly limited, see Equation (22). Without additional limitation of the predictor, this fact would lead to a wind-up effect of the integral terms when the system is saturated. This has to be avoided in the interest of an optimal estimation (in this paper, implemented by a PT1 modeling approach, Section 4.1.2).

### 4.2. Sensors and Measurands

Any information about the vehicle state is provided by the measurands. Consequently, the selection of the measured variables is a central issue in the filter design. The ROMO is equipped with sensors to determine the following variables:

- Wheel speed $\omega^{W_{i}}$
- Inertial measurement unit (IMU) fused with GPS for measuring accelerations, velocities, and angles at the center of gravity $a_{x}^{C}$, $a_{y}^{C}$, ${\dot{\psi }}^{C}$, $v^{C}$, $\beta^{C}$
- Driving and braking torque at the wheels $M_{D}^{W_{i}}$ and $M_{B}^{W_{i}}$
- Self-aligning torque at the wheels $M_{Z}^{W_{i}}$

with the index i∈{fl, fr, rl, rr} for the tire position. The ROMO is equipped with several other sensors that are not relevant for this research work, see [12]. All signals are provided by the ROMO’s vehicle dynamics control (VDC) [33]. Most of the listed variables are also used by the ESC (electronic stability control), and are thus, in principle, also available in a production vehicle. The ROMO’s sensors are analyzed in [12] with respect to the noise properties, bias, delay, etc. As a result, the sensor measurements can be represented as signals with realistic properties to synthetically generate the measurement data by simulating the reference vehicle model.

**Virtual Measurands:** In addition to the directly available measurements, the so-called virtual measurands can be used. They are calculated from an advantageous conversion of directly measurable quantities. One of the benefits of using virtual measurands is the wheel-related consideration, providing a more precise physical description of the TRFC. Another advantage is the inclusion of additional measurands in the estimation problem (driving and braking torques as well as wheel speeds). The concept of using virtual measurands for vehicle state estimation is based on [32]. For a detailed derivation, reference is, therefore, made to this. To distinguish the virtual measurands from the real ones, they are marked by a tilde.

For the calculation of the virtual measurands, the vehicle is considered as a single-track model (STM). For that reason, the axle-wise mean value of the two steering angles (left and right) can be used as a simplification $\delta_{\mathrm{STM}}^{W_{p}}=\left(\delta^{W_{i}}+\delta^{W_{i}}\right)/2$, with p∈{front, rear}, as well as the mean value of the wheel speeds on the left- and right-hand sides of the front axle $\omega_{\mathrm{STM}}^{W_{f}}=\left(\omega^{W_{fl}}+\omega^{W_{fr}}\right)/2$.

**Virtual vehicle velocity:** The virtual speed ${\tilde{v}}^{C}$ is obtained from the wheel speed $\omega_{\mathrm{STM}}^{W_{f}}$ and the effective tire radius $r_{\mathrm{eff}}$ at the front axle:

(27) $${\tilde{v}}^{C}=\omega_{\mathrm{STM}}^{W_{f}}\cdot r_{\mathrm{eff}}.$$

**Virtual longitudinal axle force:** A virtual longitudinal axle force can be determined from the driving and braking torques at the wheels and the tire radius:

(28) $${{\tilde{F}}_{x}}^{W_{p}}=\frac{M_{B}^{W_{p}}+M_{D}^{W_{p}}}{r_{\mathrm{eff}}}.$$

**Virtual lateral axle force:** The lateral acceleration at the center of gravity, the yaw rate, the virtual longitudinal axle force, and the effective steering angle are used to calculate a virtual lateral axle force:

(29) $${\tilde{F}}_{y}^{W_{f}}=\frac{1}{\cos\left(\delta_{\mathrm{STM}}^{W_{f}}\right)}\cdot \left[\frac{a_{y}^{C}\cdot l_{r}\cdot m+{\ddot{\psi }}^{C}\cdot J_{z}}{l}-\sin\left(\delta_{\mathrm{STM}}^{W_{f}}\right)\cdot {\tilde{F}}_{x}^{W_{f}}\right],\;{\tilde{F}}_{y}^{W_{r}}=\frac{1}{\cos\left(\delta_{\mathrm{STM}}^{W_{r}}\right)}\cdot \left[\frac{a_{y}^{C}\cdot l_{f}\cdot m-{\ddot{\psi }}^{C}\cdot J_{z}}{l}-\sin\left(\delta_{\mathrm{STM}}^{W_{r}}\right)\cdot {\tilde{F}}_{x}^{W_{r}}\right].$$

**Virtual side-slip angle:** The calculation of the virtual side-slip angle ${\tilde{\beta }}^{C}$ is based on the assumption that the slip angle at the front axle ${\tilde{\alpha }}_{f}$ can be approximated by the associated cornering stiffness ${\tilde{c}}_{\alpha ,f}$ as ${\tilde{\alpha }}_{f}\approx {\tilde{F}}_{y}^{W_{f}}/{\tilde{c}}_{\alpha ,f}$. The identification of the cornering stiffness ${\tilde{c}}_{\alpha ,f}$ is performed via a parameter optimization for representative driving dynamics maneuvers with DLR *MOPS*. With the help of the approximated tire-slip angle ${\tilde{\alpha }}_{f}$, the virtual side-slip angle is obtained using virtual longitudinal and lateral vehicle velocities ${\tilde{v}}_{x}^{C}$ and ${\tilde{v}}_{y}^{C}$, respectively, [32]:

(30) $${\tilde{\beta }}^{C}=\arctan\left(\frac{{\tilde{v}}_{y}^{C}}{{\tilde{v}}_{x}^{C}}\right).$$

### 4.3. Estimator Setups

The first step of estimator design is the selection of a specific filter type. In intensive investigations, a UKF with a sampling time of 20 ms has proven to be a good compromise between computational effort and accuracy. A linear Kalman filter is not an option because of strong nonlinearities. An MHE is also not taken into account due to the enormous computation time required. An extended Kalman filter (EKF) basically needs less computational effort than a UKF, but it delivers good results only for small sampling times. At the same time, a UKF provides satisfactory estimates, i.e., with a lower computational cost, even for higher sampling times.

The filter parameters, namely, the system noise and measurement noise covariance matrices Q and R, respectively, as well as the factors $\alpha_{\mathrm{UKF}}$, $\beta_{\mathrm{UKF}}$, $\gamma_{\mathrm{UKF}}$ for the approximation of the probability densities, are determined with the help of the design method presented in Section 3.3. The entries of the state vector $x\;\in {\mathbb{R}}^{4\times 1}$, which have to be estimated, are the side-slip angle, the vehicle velocity over ground, the maximum tire-road friction coefficient, and the yaw rate (see Equations (20)–(23)):

(31) $$x={\left[\begin{array}{cccc} \beta^{C} & v^{C} & \mu_{\max} & {\dot{\psi }}^{C} \end{array}\right]}^{T}.$$

The inputs $u\in {\mathbb{R}}^{11\times 1}$ consist of four-wheel steering angles, four-wheel speeds, the longitudinal and lateral acceleration, and the yaw rate:

(32) $$u={\left[\begin{array}{ccc} \delta^{W_{i}} & \omega^{W_{i}} & \begin{array}{ccc} a_{x}^{C} & a_{y}^{C} & {\dot{\psi }}^{C} \end{array} \end{array}\right]}^{T}.$$

The state $\mu_{\max}$ is constrained by the method presented in Section 2.2. According to the available sensor signals presented in the previous section, 12 variables can be measured. The measured variables y are divided into four setups for further analysis and compared with each other. The influence of different measurands on the observability of the states is assessed by the nonlinear quantitative observability measure according to Equation (12). The contribution of different measurands to the estimation is quantified in the dominance analysis according to Section 3.2 Table 1 shows the four measurement setups. The green color indicates that the respective sensor signal is used as a measurement.

Setup 1 represents the maximum configuration with all 12 available measurands. In comparison, the real side-slip angle and the real vehicle velocity are no longer available for setup 2. In setup 3, the tire self-aligning torque (SAT) is additionally removed. In Setup 4, the virtual longitudinal and lateral axle forces are not considered anymore (no driving and braking torque sensors at wheels available). With the remaining five measurands, this setup represents the minimum configuration.

### 4.4. Test Track and Maneuver

The selection of suitable excitations is of paramount importance for the analysis of an estimation filter. The excitations should contain the broadest possible spectrum of components that can arise during filter operation. In the present case, which deals with vehicle state estimation, a suitable test track or driving maneuver has to be chosen. A maneuver on a lying and crossing eight, a so-called figure-eight maneuver, is used. It covers many characteristic properties of the vehicle dynamics, and therefore, enables an optimal filter analysis:

- Entering a curve: Combined lateral and longitudinal excitation (braking and steering).
- Driving along a curve: Isolated and stationary lateral excitation (steering at a constant vehicle velocity).
- Exiting a curve: Combined lateral and longitudinal excitation (acceleration and steering).
- Straight line segment: Isolated longitudinal excitation with acceleration and braking.

**Figure 7:**
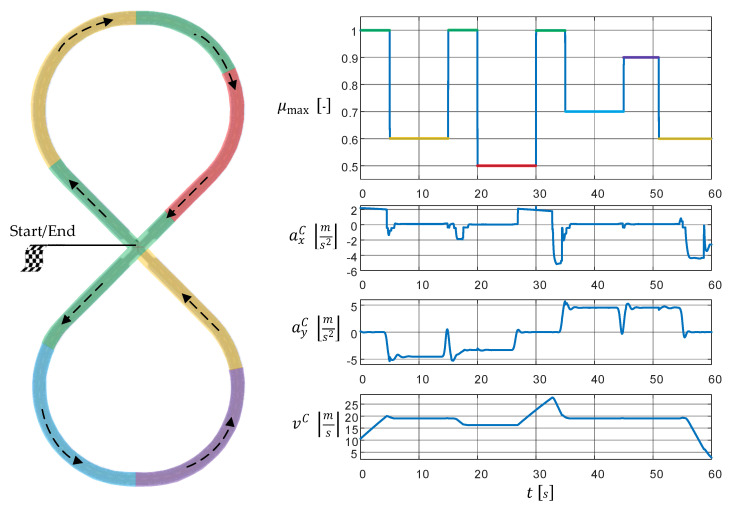
Overview of the test scenario: Test track and vehicle dynamics quantities generated by the high-fidelity reference model, see Section 4.1.1.

The track is divided into segments with different TRFCs in the range of $0.5\leq \mu_{\max}\leq 1$ (see Figure 7), which roughly correspond to driving on a road with a thin layer of ice and a dry road with a high coefficient of friction, respectively.

Further specifications of the track and the test maneuver can be found in Table 2.

## 5. Results

This section presents the results of the four filter setups mentioned above, which are parameterized and analyzed with the help of the design method according to Section 3.3. In the beginning, the results of the estimated states by a UKF are shown, i.e., the tracking performance of the filters (Section 5.1). On the one hand, the fit value is used to assess the estimation quality, i.e., to evaluate the error between estimates and true trajectories [34]. This gives a percentage fit value (100% ≙ perfect fit) and is thus a well-interpretable metric. On the other hand, the root-mean-square error (RMSE) is used as a physically interpretable error measure, see Appendix C.

Using the state-specific quantitative observability measure, observability analysis is performed in Section 5.2 for every state in each of the four setups. To compare the respective setups with each other, the following evaluation measure is defined:

(33) $$\Delta_{\mathrm{Obs},i,j}=1-\frac{\sum_{k}\sigma_{\mathrm{obs},x_{i}}\left(\mathrm{Setup}\;j\right)}{\sum_{k}\sigma_{\mathrm{obs},x_{i}}\left(\mathrm{Setup}\;4\right)}.$$

Here, the curves of the observability measure of the i^th^ state $\sigma_{\mathrm{obs},x_{i}}$ are summed up over all time steps k for every setup j and divided by the corresponding value of reference configuration 4. This provides a percentage value showing the observability improvement of setup j compared to setup 4 (minimum configuration). Next, in Section 5.3, dominance analysis is performed for each setup using the state $\mu_{\max}$ as an example to investigate the influence of the respective measurands on the estimation. The results of the dominance analysis are presented in Section 5.4 and used to rank the measurands according to their importance for the estimation. Finally, the most significant insights of the design analysis are summarized in Section 5.5.

### 5.1. Vehicle State Estimation

The estimation results are presented in Table 3 using the fit value and the RMSE for the four different filter setups for each of the estimated states.

**Figure 8:**
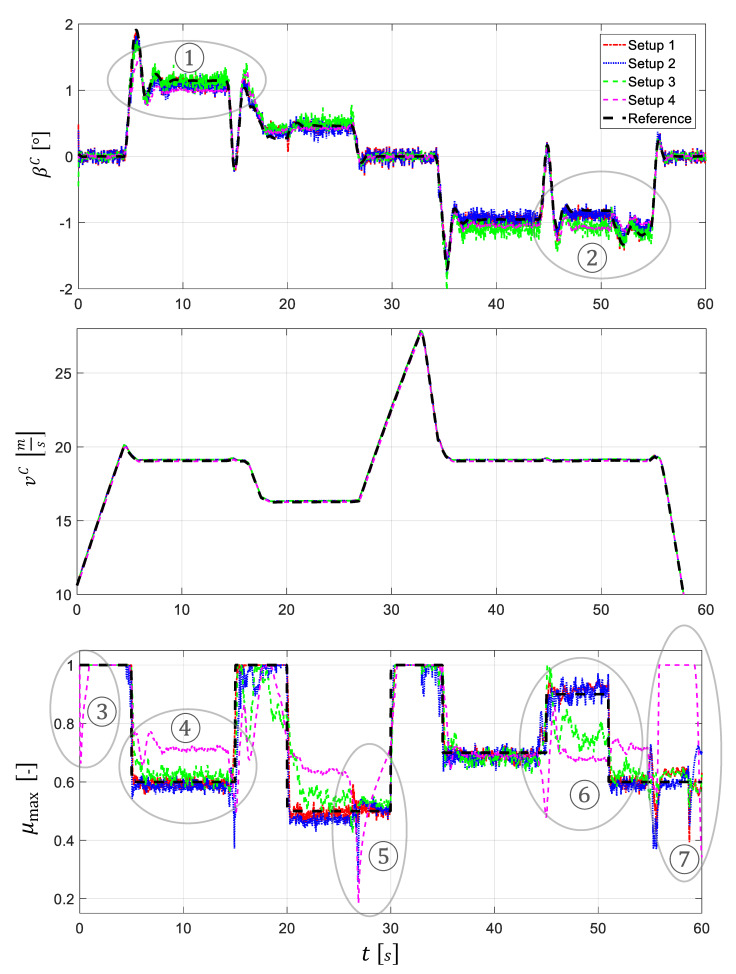
Estimated states of setup 1 to setup 4 (the estimated yaw rate is not shown).

Very good or very bad estimation results are represented by the colors green or red, respectively. Figure 8 shows the estimated trajectories corresponding to the performance values listed in Table 3 as well as the reference values.

Setup 1 (maximum configuration) can estimate all four states with a very good accuracy.

Since the side-slip angle and the vehicle velocity are measured directly, the fit values are over 90%. The TRFC can be reconstructed from the measurements very well resulting in a fit value of almost 80% or, equivalently, an RMSE of 0.04. The loss of the direct side-slip angle and vehicle velocity measurements in the second setup has only a minor effect on the estimation performance. The coefficient of friction loses almost six percentage points in the fit value compared to setup 1. However, it can be well-reconstructed thanks to the sensors, which are still available. In the third setup, in which the measurement of the tire self-aligning torque (SAT) is removed, larger changes in the estimation accuracy are observed. Nevertheless, the TRFC can be still estimated satisfactorily with a fit value of almost 60% or, equivalently, an RMSE of 0.08. For the fourth setup (minimum configuration), where the driving and braking torque information at the wheels is no longer available, the TRFC cannot be estimated satisfactorily, i.e., with a fit value of less than 15% or an RMSE of 0.15. It is noteworthy that the side-slip angle, the estimation of which is based only on the virtual measurand ${\tilde{\beta }}^{C}$, can still be well-reconstructed with a fit value of more than 80%. The vehicle velocity can be estimated by all setups identically well (fit value >97%). The yaw rate is available as a direct measurand and does not have to be reconstructed. Therefore, this state is not further examined and its time course is not shown in Figure 8. After having discussed the estimation quality in general, some specific estimation segments are analyzed in more detail below.

The individual state variables are obviously strongly coupled with each other, so that the estimation deviations over time are seen not only in a single state. In ①, the side-slip angle is heavily underestimated for setup 4, caused by an overestimation of the coefficient of friction in the same time range ④. The opposite case can be seen in ②, where the side-slip angle for setups 3 and 4 is overestimated because the corresponding coefficients of friction in ⑥ are considerably underestimated.

The observed strong coupling between the deviations in the estimated side-slip angle ${\hat{\beta }}^{C}$ and the coefficient of friction ${\hat{\mu }}_{\max}$ is also indicated, without going into further detail, by high correlation coefficients between the estimation errors of these variables. For that reason, it can be stated that a good estimate of the coefficient of friction can only be obtained by a good estimate of the side-slip angle.

In ③ and ⑤, the coefficient of friction shows for setup 4 a step-like behavior at the beginning of isolated longitudinal excitation phases (see velocity plot), converging then to a value of 1. An isolated braking maneuver ⑦ demonstrates similar behavior. For setup 4, the excitation is obviously insufficient in these regions to reconstruct the coefficient of friction from the measurements. The state is no longer observable in this case, which is also confirmed by the quantitative observability measure in the following section, and tends towards the preset friction value of 1 due to the modeled first-order dynamics of $\mu_{\max}$ (artificial stabilization, see Section 4.1.2).

### 5.2. Observability Analysis

The comparison of the observability properties of different setups (see Equation (33)) can be found in Table 4. The percentage improvement of the observability related to setup 4, with its minimum sensor equipment, is shown here for all states.

**Figure 9:**
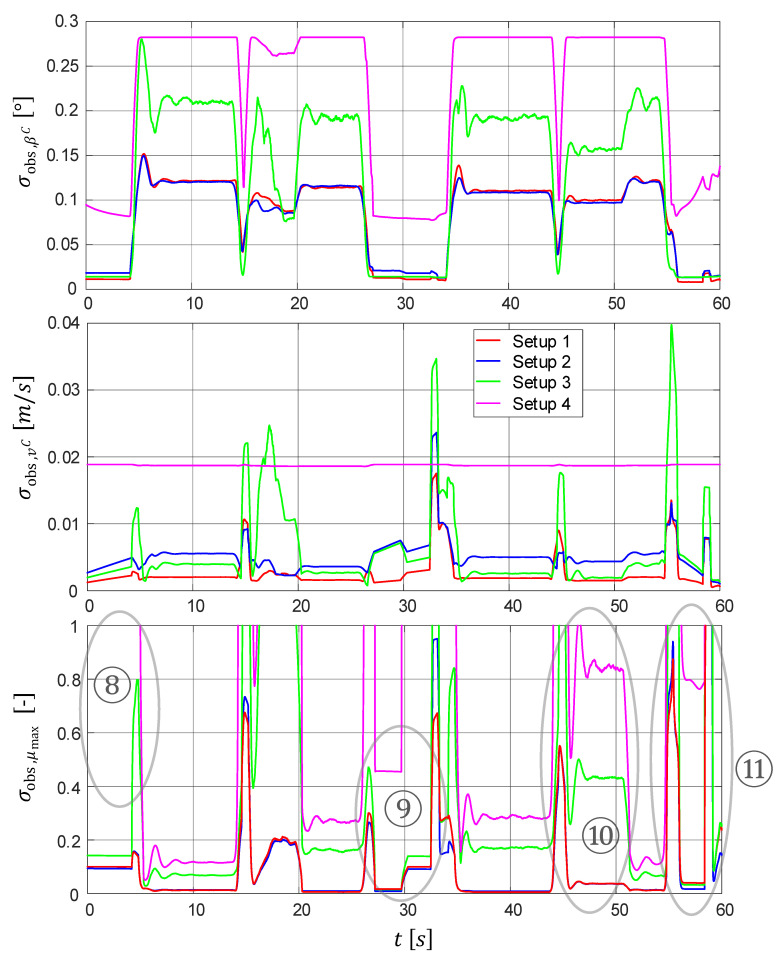
Quantitative observability measure of setup 1 to setup 4 (standard deviations of the WLS estimators, see Equation (11), with low/high values corresponding to good/poor observability).

No analysis is performed for the estimated yaw rate since this state is directly measured for each setup and is thus fully observable per se. In general, it can be seen from Table 4 that the more measurands there are available (setup 4 = minimum number of measurands, setup 1 = maximum number), the better the observability. The trajectories of the quantitative observability measure $\sigma_{obs,x_{i}}$ over time are shown in Figure 9.

**Side-slip angle** ${\hat{\beta }}^{C}$

Although the side-slip angle is directly measured in setup 1, its observability is hardly any better compared to setup 2 measuring this angle only virtually. Setup 2 leads to a large increase in observability compared to setup 3. This means that the measurand tire self-aligning torque (SAT) $M_{z}^{W_{f}}$ of setup 2 provides highly relevant information for the side-slip angle. The fact that the observability of setup 3 is by over 40% better than that of setup 4 implies that the driving and braking torques at the wheels also represent a good information source for the side-slip angle estimation.

Generally, it can be noted that setups 1 and 2 have similar and the best observability accuracies of the vehicle side-slip angle. Setup 3 provides significantly worse observability, while setup 4 delivers the worst one.

**Vehicle velocity** ${\hat{v}}^{C}$

The observability measure $\sigma_{\mathrm{obs},v}$ (see center of Figure 9) of setup 1 is with a few exceptions the smallest, which can be anticipated, since the vehicle velocity is directly measured. Peaks with poor observability are present in each setup. They appear when there is a sign change in the longitudinal vehicle acceleration (i.e., switching from acceleration to deceleration and vice versa). In these moments, there is no excitation for a short period because of the acceleration reversal. This phenomenon makes the observability worsen and is captured by the quantitative criterion. The fact that, in these moments, setup 2 is better than setup 3 implies that the additional measurand SAT contains information also about the vehicle velocity and additionally supports the estimation of ${\hat{v}}^{C}$ in such moments. Overall, the observability is very good for all setups, even for the minimally equipped setup 4, meaning that not many sensors are required when only the vehicle velocity is estimated.

**Tire road friction coefficient** ${\hat{\mu }}_{max}$

The observabilities of setups 1 and 2 are nearly identical. In both cases, the observability is improved by over 96% compared to the reference setup. This fact implies that the measurands’ side-slip angle and vehicle velocity, which can only be found in setup 1, do not yield in this case any benefits in terms of observability.

The observability of setup 3 is worse than that of setup 1 or 2 because of missing measurands required for maneuver segments with lateral excitation (namely, $\beta^{C}$, $v^{C},$ and $M_{z}^{W_{f}}$). In contrast, segments with longitudinal excitation—such as, e.g., ⑨—demonstrate a similarly good observability accuracy as setups 1 and 2. This indicates that the sensor signals driving and braking torques $M_{D}^{W_{i}}$ and $M_{B}^{W_{i}}$, respectively, contain enough information about the TRFC in this case.

In ⑩, setups 3 and 4 deliver worse observability than setups 1 and 2. This phenomenon is also reflected in the estimation accuracy shown in ⑥ (see Figure 8), where significant underestimation is present in both setups. In maneuver segments with isolated longitudinal excitation, namely ⑧, ⑨ (accelerating), and ⑪ (braking), the total loss of observability for setup 4 is captured by the observability measure when $\sigma_{\mathrm{obs},\mu_{\max}}\geq 1$. In the corresponding time plots of the estimated TRFC in Figure 8, namely ③, ⑤, and ⑦, $\mu_{\max}$ converges to its default stabilized value of 1 due to lack of observability.

### 5.3. Dominance Analysis

The measurands were analyzed with respect to their observability property in the previous subsection, and their contributions to the estimation are presented now in what follows (dominance properties). Due to the focus of the paper, the dominance analysis is limited to the state TRFC $\mu_{\max}$. However, the methodology would also allow analysis to be performed of other states as well. Table 5 shows the contributions of the measurands to the estimation of the state $\mu_{\max}$ for different setups.

Although a total of 12 measurands are available in setup 1, only three measurands—namely, ${\tilde{F_{x}}}^{W_{f}}\;\mathrm{and}\;{\tilde{F_{x}}}^{W_{r}}$(i.e., driving and braking torque $M_{D}^{W_{i}}$ and $M_{B}^{W_{i}}$, respectively) and the SAT $M_{z}^{W_{f}}—$are used by the filter to reconstruct $\mu_{\max}$ and account for almost 90% of the dominance. The measurements of the side-slip angle $\beta^{C}$ and the vehicle velocity $v^{C}$ appear, at first glance, to be very valuable, with a negligible contribution of less than 5% in total. The dominance of the three measurands mentioned above does not change for setup 2. The removal of $\beta^{C}$ and $v^{C}$ seems to be compensated by the virtual axle lateral force ${\tilde{F_{y}}}^{W_{f}}$.

In setup 3, where one of the dominant measurands, the SAT $M_{z}^{W_{f}}$ (30% of the total dominance), is no longer available, the contribution of the remaining sensors changes. One part of the estimation contribution of $M_{z}^{W_{f}}$ seems to be compensated by the virtual axle lateral forces, and another part by the measurement of the vehicle lateral acceleration $a_{y}^{C}$. At the same time, part of the contribution of the virtual axle forces ${\tilde{F_{x}}}^{W_{j}}$ is taken over by the longitudinal acceleration $a_{x}^{C}$. However, the measurands ${\tilde{F_{x}}}^{W_{j}}$ still remain dominant. The virtual side-slip angle makes a small contribution to the TRFC estimate for the first time in setup 3.

In setup 4, all previously dominant measurands are no longer available. The virtual side-slip angle ${\tilde{\beta }}^{C}$ and the vehicle lateral acceleration $a_{y}^{C}$ take over the dominance of the sensors with a total contribution of more than 80%. The virtual vehicle velocity ${\tilde{v}}^{C}$ as well as the longitudinal acceleration $a_{x}^{C}$ account for less than 10% of the overall dominance. As shown in the previous section, the problem is not observable for large parts of longitudinal maneuvers in setup 4. For that reason, it is completely plausible that no information can be extracted from the corresponding measurands.

For all setups, it seems that the yaw rate ${\dot{\psi }}^{C}$ is involved in the estimation of the TRFC with a relatively small contribution. However, it should be noted that ${\dot{\psi }}^{C}$ is used to calculate the virtual lateral axle forces ${\tilde{F_{y}}}^{W_{j}}$ as well as the virtual side-slip angle ${\tilde{\beta }}^{C}$ resulting in its additional indirect contribution through these variables.

### 5.4. Measurands Ranking

Based on the dominance analysis of the four different setups performed in the previous section (see Table 5), the influence and thus the “value” of each measurand for estimating the TRFC can be assessed. In Table 6, a ranking of the measurands is given.

The measurands with the biggest contribution to the TRFC estimation are the virtual longitudinal axle forces calculated from the wheel driving and braking torques. They are followed by the tire self-aligning torque (SAT). The sensor signals from the IMU, i.e., accelerations and velocities, are ranked next. The virtual side-slip angle and virtual vehicle velocity share last place in the ranking. Due to their purely approximate calculation (${\tilde{v}}^{C}$ with uncertainty in the tire radius, see Equation (27), ${\tilde{\beta }}^{C}$ with uncertainty in the cornering stiffness approximation, see Equation (30)), they are used more as additional sources. The information from directly available sensor signals, i.e., real ones, should be preferred. When looking at the table sequence, it is noticeable that information about the coefficient of friction is extracted from (listed in descending order)

- forces or moments
- accelerations (including virtual lateral axle forces since they are calculated from these measurands)
- velocity or rotational speeds
- virtual measurands ${\tilde{v}}^{C}$, ${\tilde{\beta }}^{C}$.

Since the tire forces and moments represent the most precise physical descriptor of the TRFC in terms of causality, they contain the most information about this state. For other descriptors, (inaccurate) conversion is necessary. It could be a purely analytical relationship (e.g., a=F/m) or an integration (e.g., v=∫a).

### 5.5. Summary of the Design Analysis

The side-slip angle could be estimated quite well with a total fit value of over 80% with all setups, even with those that only have virtual measurands and IMU information. The vehicle velocity could be reconstructed very well by all setups with a fit value of 97%. Therefore, it can be stated that no additional complex sensor technology is required to estimate this state. The coefficient of friction between the tire and the road is the state that is most difficult to reconstruct. The removal of sensors, starting from setup 1, shows large, direct effects on the estimation accuracy and observability properties.

The wheel driving or braking torques$—M_{D}^{W_{i}}$ and $M_{B}^{W_{i}}$, respectively—and the tire self-aligning torque $M_{z}^{W_{f}}$ have proven to be the most valuable measurands for estimating the TRFC. All of them lead to very good observability and represent dominant measurands for reconstructing the coefficient of friction. The torques $M_{D}^{W_{i}}$ and $M_{B}^{W_{i}}$ seem to be important for information extraction from longitudinal excitations, and the SAT $M_{z}^{W_{f}}$ for lateral excitations. In this case, measurements of the side-slip angle and vehicle velocity do not provide any significant advantage. Therefore, the considerable effort required to provide these measurands can be saved in the case of production vehicles.

A setup that only uses the basic information from the IMU (setup 4) provides unsatisfactory results for TRFC estimation (approx. 15% fit value). For maneuvers with higher excitation, better estimation results are possible since the problem is then more observable. The estimation errors of the side-slip angle and TRFC are correlated, so that a good estimate of the TRFC is only possible if there is a good estimate of the side-slip angle.

## 6. Conclusions

In this paper, a novel and universal design and analysis method for nonlinear Kalman filters was presented. The method allows a systematic investigation of the measurands’ influence on the estimation problem in terms of

- Observability properties: Two novel quantitative nonlinear observability measures were presented.
  ○ Evaluation of the overall system via the numerical condition number of the observability matrix.
  ○ A state-specific and physically interpretable observability measure via a weighted least-squares approach.
- Dominance properties: A new method quantifying the contribution and information content of a measurand for the state reconstruction.

To determine an optimal filter parameterization, the method uses an optimization algorithm. As an example, the method was applied to a vehicle state estimation problem focusing on the coefficient of friction between the tire and the road. For this purpose, an unscented Kalman filter with constraints was used. A nonlinear two-track model served as the prediction model, while a high-accuracy Modelica multi-body model was used as the reference model. The TRFC, whose estimation was the most challenging one among all states, could be reconstructed with a fit value lying between 15% and 80%. Using the design method, four filter setups with different available sensors (from a maximum to a minimum number of them) were analyzed and compared. Here, it was shown that for the structural design of an estimation filter, it is worth doing preliminary investigations about the influence of the measurands. For this estimation problem, it could be identified, for example, that the measurements of the side-slip angle and vehicle velocity over ground, which are costly to provide in a production vehicle, have a negligible influence on the investigated estimation properties for certain setups. Both sensors possess a total contribution to the estimation (dominance) of less than 5%. Thus, a sensor for measuring these signals is not necessary.

We plan to perform the analysis with additional sensors in the future, especially with new types of sensors such as camera, radar, and lidar. The vehicle state estimation problem should be implemented with the most promising setups in real driving tests, both prototypically and on an ECU (embedded system).

## Figures and Tables

**Table 1:** Overview of the measurands for each of the estimator setups.

Measurand	${\tilde{\beta }}^{C}$	${\tilde{v}}^{C}$	${\tilde{F_{y}}}^{W_{f}}$	${\tilde{F_{y}}}^{W_{r}}$	${\tilde{F_{x}}}^{W_{f}}$	${\tilde{F_{x}}}^{W_{r}}$	$M_{z}^{W_{f}}$	$a_{x}^{C}$	$a_{y}^{C}$	${\dot{\psi }}^{C}$	$\beta^{C}$	$v^{C}$
**Setup**												
1												
2												
3												
4												

**Table 2:** Overview of the test maneuver specifications.

Value	Min	Max
TRFC $\mu_{\max}\;\left[-\right]$	0.5	1
Vehicle velocity $v^{C}\;\left[m/s\right]$	2.5	28
Longitudinal acceleration $a_{x}^{C}\;\left[m/s^{2}\right]$	−5.2	2.2
Lateral acceleration $a_{y}^{C}\;\left[m/s^{2}\right]$	−5.3	5.7
Track length s [m]	1100	

**Table 3:** Overview of the estimation error quantities of the four states for each setup.

Criteria	Fit [%]				RMSE			
State	${\hat{\beta }}^{C}$	${\hat{v}}^{C}$	${\hat{\mu }}_{max}$	${\hat{\dot{\psi }}}^{C}$	${\hat{\beta }}^{C}\;\left[{}^{{}^{\circ}}\right]$	${\hat{v}}^{C}\;\left[m/s\right]$	${\hat{\mu }}_{max}\;\left[-\right]$	${\hat{\dot{\psi }}}^{C}\;\left[{}^{{}^{\circ}}\right]$
**Setup**								
1	90.7	97.5	79.6	99.9	0.07	0.09	0.04	0.01
2	89.4	97.5	73.8	99.9	0.08	0.09	0.05	0.01
3	84.1	97.5	59.0	99.9	0.12	0.09	0.08	0.01
4	82.9	97.1	14.9	86.5	0.13	0.09	0.15	1.50

**Table 4:** Overview of the states’ observability improvement compared to setup 4.

	$\Delta_{Obs}(\mathrm{Setup}\;4,\;\mathrm{Setup}\;i)$ [%]			
State	${\hat{\beta }}^{C}$	${\hat{v}}^{C}$	${\hat{\mu }}_{\max}$	${\hat{\dot{\psi }}}^{C}$
**Setup**				
1	63.1	85.9	96.4	-
2	61.1	72.7	96.4	-
3	41.8	68.6	87.53	-
4	Reference			

**Table 5:** Overview of the measurands’ dominance for the TRFC estimation.

	Contribution ${\bar{\gamma }}_{\mu_{max}}$ $y_{i}\to \mu_{max}\;\left[\%\right]$											
Measurand	${\tilde{\beta }}^{C}$	${\tilde{v}}^{C}$	${\tilde{F_{y}}}^{W_{f}}$	${\tilde{F_{y}}}^{W_{r}}$	${\tilde{F_{x}}}^{W_{f}}$	${\tilde{F_{x}}}^{W_{r}}$	$M_{z}^{W_{f}}$	$a_{x}^{C}$	$a_{y}^{C}$	${\dot{\psi }}^{C}$	$\beta^{C}$	$v^{C}$
**Setup**												
1	≈0	0.2	0.3	1.7	34.7	22.6	31.4	2.1	1.5	0.8	0.5	4.2
2	≈0	2.8	8.9	≈0	32.5	25.0	29.8	≈0	0.8	0.2	-	-
3	3.7	2.0	16.0	0.9	16.5	29.1	-	17.5	13.5	0.8	-	-
4	45.3	2.8	-	-	-	-	-	6.3	37.8	7.8	-	-

**Table 6:** Measurands’ ranking based on their importance for the TRFC estimation.

Rank	Measurement	Equation No.
1	${\tilde{F_{x}}}^{W_{f}}$, ${\tilde{F_{x}}}^{W_{r}}$ (i.e., $M_{D}^{W_{i}}$, $M_{B}^{W_{i}}$)	(28)
2	$M_{z}^{W_{f}}$	(A21)
3	$a_{x}^{C}$, $a_{y}^{C}$, ${\tilde{F_{y}}}^{W_{f}}$, ${\tilde{F_{y}}}^{W_{r}}$	(A4), (29)
4	$v^{C}$, ${\dot{\psi }}^{C}$	(21), (23)
5	$\beta^{C}$	(20)
6	${\tilde{v}}^{C}$, ${\tilde{\beta }}^{C}$	(27), (30)

## Data Availability

Not applicable.

## Lists of Symbols, Nomenclature and Abbreviations

**Formula symbols**	**Unit**	**Description**
$\beta^{C}$	[rad]	Vehicle’s side slip angle
$v^{C}$	[m/s]	Vehicle’s velocity over ground
${\dot{\psi }}^{C}$	[rad/s]	Vehicle’s yaw rate
$M_{z}^{W_{i}}$	[N]	Tire self-aligning torque
$\omega^{W_{i}}$	[rad/s]	Wheel speed
$\delta^{W_{i}}$	[rad]	Wheel steering angle
$\mu_{\max}$	[−]	Coefficient of friction between tire and road
**Abbreviations**	**Explanation**	
DLR	German Aerospace Center	
EKF/UKF	Extended/Unscented Kalman filter	
FMU/FMI	Functional mockup unit/interface	
IMU	Inertial measurement unit	
MHE	Moving horizon estimation/estimator	
MOPS	Multi-objective parameter synthesis (DLR optimization tool)	
ROMO	short for ROboMObil—DLR’s robotic electric vehicle	
SAT	Tire self-aligning torque	
STM	Single track model	
TRFC	Tire road friction coefficient	
WLS	Weighted least squares	
**Nomenclatures**	**Explanation**	
${\left(\cdot \right)}^{W_{i}}$	Quantity expressed in the i-th wheel robot coordinate system	
${\left(\cdot \right)}^{C}$	Quantity expressed in the car coordinate system with origin in CoG	
$\tilde{\left(\cdot \right)}$	Virtual measurand	
$\hat{\left(\cdot \right)}$	Estimated state	

## Appendix A. Two-Track Model

For the equations of the two-track model given below, reference is also made to Figure 5. The air resistance forces are given by:

(A1) $$F_{\mathrm{Air},x}^{C}=\frac{1}{2}c_{w}\;\rho \;A_{x}v^{C}{}^{2}\cos\left(\beta^{C}\right),\;F_{\mathrm{Air},y}^{C}=\frac{1}{2}c_{w}\;\rho \;A_{x}v^{C}{}^{2}\sin\left(\beta^{C}\right).$$

The longitudinal and lateral forces at the center of gravity are determined by:

(A2) $$\begin{array}{l} F_{x}^{C}=-F_{y}^{W_{fl}}\sin\left(\delta^{W_{fl}}\right)-F_{y}^{W_{fr}}\sin\left(\delta^{W_{fr}}\right)-F_{y}^{W_{rl}}\sin\left(\delta^{W_{rl}}\right)-F_{y}^{W_{rr}}\sin\left(\delta^{W_{rr}}\right)\\ \;\;\;\;\;+F_{x}^{W_{fl}}\cos\left(\delta^{W_{fl}}\right)+F_{x}^{W_{fr}}\cos\left(\delta^{W_{fr}}\right)+F_{x}^{W_{rl}}\cos\left(\delta^{W_{rl}}\right)\\ \;\;\;\;\;+F_{x}^{W_{rr}}\cos\left(\delta^{W_{rr}}\right)-F_{\text{Air},x}^{C} \end{array}$$

(A3) $$\begin{array}{l} F_{y}^{C}=F_{y}^{W_{fl}}\cos\left(\delta^{W_{fl}}\right)+F_{y}^{W_{fr}}\cos\left(\delta^{W_{fr}}\right)+F_{y}^{W_{rl}}\cos\left(\delta^{W_{rl}}\right)+F_{y}^{W_{rr}}\cos\left(\delta^{W_{rr}}\right)\\ \;\;\;\;\;+F_{x}^{W_{fl}}\sin\left(\delta^{W_{fl}}\right)+F_{x}^{W_{fr}}\sin\left(\delta^{W_{fr}}\right)+F_{x}^{W_{rl}}\sin\left(\delta^{W_{rl}}\right)+F_{x}^{W_{rr}}\sin\left(\delta^{W_{rr}}\right)\\ \;\;\;\;\;-F_{\text{Air},y}^{C}. \end{array}$$

The accelerations at the vehicle center of gravity are calculated as follows:

(A4) $$a_{x}^{C}=\frac{F_{x}^{C}}{m},\;\;\;\;\;a_{y}^{C}=\frac{F_{y}^{C}}{m}.$$

The quasi-stationary wheel loads are geometrically calculated from the lateral and longitudinal acceleration at the vehicle center of gravity:

(A5) $$F_{z}^{W_{fl}}=m\left(\frac{l_{r}}{l}g-\frac{h_{c}}{l}a_{x}^{C}\right)\left(\frac{1}{2}-\frac{h_{c}}{b_{f}g}a_{y}^{C}\right),\;F_{z}^{W_{fr}}=m\left(\frac{l_{r}}{l}g-\frac{h_{c}}{l}a_{x}^{C}\right)\left(\frac{1}{2}+\frac{h_{c}}{b_{f}g}a_{y}^{C}\right),\;F_{z}^{W_{rl}}=m\left(\frac{l_{f}}{l}g+\frac{h_{c}}{l}a_{x}^{C}\right)\left(\frac{1}{2}-\frac{h_{c}}{b_{r}g}a_{y}^{C}\right),\;F_{z}^{W_{rr}}=m\left(\frac{l_{f}}{l}g+\frac{h_{c}}{l}a_{x}^{C}\right)\left(\frac{1}{2}+\frac{h_{c}}{b_{r}g}a_{y}^{C}\right).$$

## Appendix B. Tire Model

For the tire model, a slightly simplified version of Pacejka’s Magic Formula 5.2 [30] is used. As a simplification, among others, the wheel camber is neglected ($\gamma^{W_{i}}=0$), all lambda scaling values are chosen to be λ=1 (except for the coefficient of friction dependent factors $\lambda_{\mu x}=\lambda_{\mu y}=\mu_{\max}$), and the transient tire behavior is neglected. The tire position is described by the index i∈{fl, fr, rl, rr}.

The wheel load-dependent tire radius is:

(A6) $$R^{W_{i}}=R_{0}^{W_{i}}-\rho_{i,0}\left(D_{\mathrm{roll}}\mathrm{atan}\left(B_{roll}\frac{\rho_{i}}{\rho_{i,0}}\right)+F_{roll}\frac{\rho_{i}}{\rho_{i,0}}\right).$$

The longitudinal tire slip:

(A7) $$s_{x}^{W_{i}}=-\frac{v_{x}^{W_{i}}-\omega^{W_{i}}R^{W_{i}}}{\left|v_{x}^{W_{i}}\right|}.$$

The wheel load increment:

(A8) $$\Delta F_{z}^{W_{i}}=\frac{F_{z}^{W_{i}}-F_{z0}^{W_{i}}}{F_{z0}^{W_{i}}}.$$

The lateral tire slip:

(A9) $$s_{y}^{W_{fl}}=\tan\left(\alpha^{W_{fl}}\right)+S_{Hy}^{W_{fl}},\;s_{y}^{W_{fr}}=-\tan\left(\alpha^{W_{fr}}\right)+S_{Hy}^{W_{fr}},\;s_{y}^{W_{rl}}=\tan\left(\alpha^{W_{rl}}\right)+S_{Hy}^{W_{rl}},\;s_{y}^{W_{rr}}=-\tan\left(\alpha^{W_{rr}}\right)+S_{Hy}^{W_{rr}}.$$

**Pure longitudinal tire forces**

(A10) $$F_{x0}^{W_{i}}=D_{x}^{W_{i}}\sin\left(C_{x}\mathrm{atan}\left(B_{x}^{W_{i}}s_{x}^{W_{i}}-E_{x}^{W_{i}}\left(B_{x}^{W_{i}}s_{x}^{W_{i}}-\mathrm{atan}\left(B_{x}^{W_{i}}s_{x}^{W_{i}}\right)\right)\right)\right)+S_{Vx}^{W_{i}},$$

(A11) $$E_{x}^{W_{i}}=\left(p_{Ex1}+p_{Ex2}\Delta F_{z}^{W_{i}}+p_{Ex3}\Delta F_{z}^{{W_{i}}^{2}}\right)\left(1-p_{Ex4}sgn\left(s_{x}^{W_{i}}\right)\right),$$

(A12) $$D_{x}^{W_{i}}=\left(p_{Dx1}+p_{Dx2}\Delta F_{z}^{W_{i}}\right)\mu_{\max},\;K_{x}^{W_{i}}=F_{z}^{W_{i}}\left(p_{Kx1}+p_{Kx2}\Delta F_{z}^{W_{i}}\right)e^{\left(p_{Kx3}\Delta F_{z}^{W_{i}}\right)},$$

(A13) $$B_{x}^{W_{i}}=\frac{K_{x}^{W_{i}}}{C_{x}D_{x}^{W_{i}}},\;C_{x}=p_{Cx1},\;S_{Vx}^{W_{i}}=F_{z}^{W_{i}}\left(p_{Vx1}+p_{Vx2}\Delta F_{z}^{W_{i}}\right)\mu_{\max}.$$

**Pure lateral tire forces**

(A14) $$F_{y0}^{W_{i}}=D_{y}^{W_{i}}\sin\left(C_{y}\mathrm{atan}\left(B_{y}^{W_{i}}s_{y}^{W_{i}}-E_{y}^{W_{i}}\left(B_{y}^{W_{i}}s_{y}^{W_{i}}-\mathrm{atan}\left(B_{y}^{W_{i}}s_{y}^{W_{i}}\right)\right)\right)\right)+S_{Vy}^{W_{i}},$$

(A15) $$D_{y}^{W_{i}}=\left(p_{Dy1}+p_{Dy2}\Delta F_{z}^{W_{i}}\right)\mu_{\max},$$

(A16) $$E_{y}^{W_{i}}=\left(p_{Ey1}+p_{Ey2}\Delta F_{z}^{W_{i}}\right)\left(1-p_{Ey3}sgn\left(s_{y}^{W_{i}}\right)\right),$$

(A17) $$K_{y}^{W_{i}}=p_{Ky1}F_{z0}\sin\left(2\mathrm{atan}\left(\frac{F_{z}^{W_{i}}}{p_{Ky2}F_{z0}}\right)\right),$$

(A18) $$B_{y}^{W_{i}}=\frac{K_{y}^{W_{i}}}{C_{y}D_{y}^{W_{i}}},\;C_{y}=p_{Cy1},$$

(A19) $$S_{Hy}=p_{Hy1}+p_{Hy2}\;\Delta F_{z}^{W_{i}},\;S_{Vy}=F_{z}^{W_{i}}\left(p_{Vy1}+p_{Vy2}\Delta F_{z}^{W_{i}}\right)\mu_{\max}$$

**Combined tire forces:** Furthermore, the influence of a combined slip is taken into account for the longitudinal and lateral forces by multiplying the tire forces for a pure slip from Equation (A10) and Equation (A14) with a weighting function dependent on the total slip [30]:

(A20) $$F_{x}^{W_{i}}=F_{x0}^{W_{i}}\cdot G_{x,s_{y}}^{W_{i}},\;F_{y}^{W_{i}}=F_{y0}^{W_{i}}\cdot G_{y,s_{x}}^{W_{i}}.$$

**Tire self-aligning torque**

(A21) $$M_{z0}^{W_{i}}=-t^{W_{i}}\;F_{yo}^{W_{i}}+M_{zr}^{W_{i}}.$$

The pneumatic trail:

(A22) $$t^{W_{i}}=D_{t}^{W_{i}}\cos\left(C_{t}^{W_{i}}\mathrm{atan}\left(B_{t}^{W_{i}}\;\alpha_{t}^{W_{i}}-E_{t}^{W_{i}}\left(B_{t}^{W_{i}}\;\alpha_{t}^{W_{i}}-\mathrm{atan}\left(B_{t}^{W_{i}}\;\alpha_{t}^{W_{i}}\right)\right)\right)\right)\cos\left(\alpha^{W_{i}}\right),$$

(A23) $$\alpha_{t}^{W_{i}}=\alpha^{W_{i}}+S_{Ht}^{W_{i}}.$$

The residual torque:

(A24) $$M_{zr}^{W_{i}}=D_{r}^{W_{i}}\cos\left(\mathrm{atan}\left(B_{r}^{W_{i}}\alpha_{r}^{W_{i}}\right)\right)\cos\left(\alpha^{W_{i}}\right),\;\alpha_{r}^{W_{i}}=\alpha^{W_{i}}+S_{Hf}^{W_{i}},$$

(A25) $$S_{Hf}^{W_{i}}=S_{Hy}^{W_{i}}+\frac{S_{Vy}^{W_{i}}}{K_{y}^{W_{i}}},\;B_{t}^{W_{i}}=\frac{\left(q_{Bz1}+q_{Bz2}\Delta F_{z}^{W_{i}}+q_{Bz3}\Delta F_{z}^{{W_{i}}^{2}}\right)}{\mu_{\max}}$$

(A26) $$C_{t}^{W_{i}}=q_{Cz1},\;D_{t}^{W_{i}}=F_{z}^{W_{i}}\left(q_{Dz1}+q_{Dz2}\;\Delta F_{z}^{W_{i}}\right)\frac{R_{w0}}{F_{z0}},$$

(A27) $$E_{t}^{W_{i}}=q_{Ez1}+q_{Ez2}\;\Delta F_{z}^{W_{i}}+q_{Ez3}\;\Delta F_{z}^{{W_{i}}^{2}},\;S_{Ht}^{W_{i}}=q_{Hz1}+q_{Hz2}\;\Delta F_{z}^{W_{i}},$$

(A28) $$B_{r}^{W_{i}}=\frac{q_{Bz9}}{\mu_{\max}}+q_{Bz10}\;B_{y}^{W_{i}}C_{y},\;D_{r}^{W_{i}}=F_{z}^{W_{i}}\left(q_{Dz6}+q_{Dz7}\;\Delta F_{z}^{W_{i}}\right).$$

## Appendix C. Error Measures

The root-mean-square error (RMSE) between a reference vector $x_{\mathrm{ref},\;i}$ and a vector ${\hat{x}}_{i}$ with n sample points is defined as:

(A29) $$\mathrm{RMSE}=\sqrt{\frac{1}{n}\sum_{i=1}^{n}{\left({\hat{x}}_{i}-x_{\mathrm{ref},\;i}\right)}^{2}}.$$

The percentage fit value is calculated as:

(A30) $$\mathrm{Fit}\;\left[\%\right]=\left(1-\frac{\sqrt{\sum_{i=1}^{n}{\left({\hat{x}}_{i}-x_{\mathrm{ref},\;i}\right)}^{2}}}{\sqrt{\sum_{i=1}^{n}{\left(x_{\mathrm{ref},i}-\frac{1}{n}\sum_{i=1}^{n}x_{\mathrm{ref},i}\right)}^{2}}}\right)\cdot 100.$$
